# SEFormer for medical image segmentation with integrated global and local features

**DOI:** 10.1038/s41598-025-25450-1

**Published:** 2025-11-24

**Authors:** Chen Ge, Haoze Pan, Yihua Song, Xia Zhang, Zuojian Zhou

**Affiliations:** 1 School of Artificial Intelligence and Information Technology, Nanjing University of Chinese Medicine, Nanjing, 210023 China; 2 Shandong University of Engineering and Vocational Technology, Jinan, 250200 China; 3 Jiangsu Province Engineering Research Center of TCM Intelligence Health Service, Nanjing University Of Chinese Medicine, Nanjing, 210023 China

**Keywords:** Computational biology and bioinformatics, Medical research

## Abstract

This paper proposes a novel medical image segmentation method, SEFormer, which effectively leverages both local and global features representations to enhance segmentation accuracy and efficiency. To address the limitations of existing image segmentation methods based on transformers and CNNs cannot in simultaneously capturing both local and global features, we propose a novel hybrid network architecture that combines SENet, CNNs( Specifically ResNet), and Transformer. In this design, SENet is employed to enhance the global feature representation capabilities of CNNs, while the Transformer component compensates for the limitations of CNNs in capturing local and channel-specific features. In addition, to prevent feature loss during feature extraction, we draw inspiration from the concept of image pyramid models, obtain a larger receptive field, and use SE fusion to fuse local and global features in each layer of the feature pyramid, ensuring a more comprehensive and complete representation of image information. In the image segmentation task on the CHASEDB dataset, our method achieves great performance, improving the segmentation accuracy by 3.25% compared to existing methods.

## Introduction

The Semantic segmentation is a computer vision task that assigns a class label to pixels using a deep learning algorithm. It helps computers understand visual information and distinguish different object classes and background regions in an image. Semantic segmentation is especially useful for medical image analysis, as it can help doctors diagnose diseases, plan surgeries, and monitor treatments CNNs^1^are a popular choice for semantic segmentation, as they can extract features from images and learn complex patterns. However, CNNs also have some limitations, such as limited receptive fields, inductive bias, and excessive reliance on local information. To overcome these limitations, some researchers have explored the use of transformers, which are originally designed for natural language processing, to process visual data. Transformers can capture global dependencies and long-range interactions between pixels, which are beneficial for semantic segmentation^2^. One of the most influential transformer models for computer vision is Vision Transformer (ViT)^3^, which divides an image into patches and treats them as tokens. ViT applies a standard transformer encoder to the patch tokens and outputs a sequence of feature vectors. ViT can achieve competitive results with CNNs on image classification tasks, but it requires a large amount of data and computational resources.

In CNNs network models designed for vision tasks, the architecture proposed by ResNet has demonstrated remarkable effectiveness in extracting image features.ResNet (Residual Networks) is a type of deep convolutional neural network (CNN) architecture proposed by researchers from Microsoft Research, including Kaiming He, in 2015^4^ . The design goal of ResNet is to address the issues of vanishing gradients and exploding gradients in deep neural networks, enabling the training of very deep neural networks.The key innovation of ResNet is the use of residual blocks, which incorporate a mechanism known as “residual learning.” In traditional neural networks, deep networks are constructed by stacking multiple layers, but as the network depth increases, the performance may degrade due to the difficulty of training extremely deep networks. ResNet introduces residual blocks, allowing the network to directly learn residuals (remaining information) instead of attempting to learn the original mapping. With the help of this mechanism, ResNet enables the model to more easily learn the identity mapping, while the non-linear mapping focuses on learning the residual. This architecture mitigates the problem of vanishing gradients with increasing depth, allowing the construction of very deep networks. And it has achieved significant success in tasks such as image classification, object detection, semantic segmentation, becoming one of the classic models in the field of deep learning. However, ResNet still has shortcomings in extracting global features from images.

Swin Transformer, a variant of ViT, enhances the efficiency and scalability of the original architecture^5^. It adopts a hierarchical structure composed of multiple stages, each containing multiple layers. A key innovation of Swin Transformer is the introduction of a window-based self-attention mechanism, which significantly reduces the computational complexity and memory consumption of the standard self-attention. To further expand the the receptive field and enrich feature diversity, a shifted window strategy to is employed across layers. Benefiting from these design choices, Swin Transformer achieves state-of-the-art results on various vision tasks, including semantic segmentation. However, it still comes with certain drawbacks. Notably, its relatively large model size contributes to heightened computational complexity and memory requirements, particularly when handling high-resolution images. The model’s substantial parameter count demands ample data for training to avoid overfitting, and the associated challenges in loading and storing these parameters may arise on resource-constrained devices. Additionally, the intricate structure of Swin Transformer may reduce interpretability compared to traditional convolutional neural networks, diminishing our ability to discern the specific roles of each attention head. Furthermore, the transferability of Swin Transformer models may not match that of some classic convolutional architectures across diverse tasks and datasets. It’s crucial to consider these limitations in the context of specific use cases and task requirements.

SENet (Squeeze-and-Excitation Network) is a deep neural network architecture that aims to improve the performance of models on tasks such as image classification^6^. It was proposed by Jie Hu, Li Shen and others in 2017, and achieved significant performance improvement on the ImageNet dataset. By introducing a mechanism called “Squeeze-and-Excitation”, it automatically learns the inter-channel relationships, so as to allocate the network’s attention more effectively. The design of SENet enables the network to adaptively focus on the importance of different channels in the input image, thereby enhancing the model’s representation ability. Due to its simple and effective idea, SENet has achieved good results in image classification and other fields. In the field of computer vision, SENet employs a squeeze-and-excitation mechanism designed to enhance model performance by reinforcing channel relationships. However, when applied as a standalone model for certain visual tasks, SENet’s performance is not as satisfactory. Typically, SENet is more commonly regarded as an effective channel attention mechanism and is often embedded within larger network architectures to achieve efficient feature fusion and overall performance improvement. This suggests that when used independently for specific tasks, SENet’s effectiveness is relatively limited, and its true value is realized when collaboratively employed with other modules.

While existing models such as the Swin Transformer have made significant progress in balancing efficiency and performance for semantic segmentation, challenges remain in fully capturing both local and global features in a unified framework. To address these limitations, we propose SEFormer, a novel architecture for semantic segmentation that integrates the strengths of both CNNs and the Swin Transformer. It adopts a dual-tower network design, with one branch built upon the Swin Transformer and the other based on CNNs. To further enhance feature representation, SENet modules are incorporated into both branches, enabling adaptive recalibration of channel-wise feature responses by modeling inter-channel dependencies. Additionally, a skip connection strategy is employed to effectively fuse local and global information from the two branches. To capture multi-scale contextual information and enlarge the receptive field, SEFormer also integrates an image pyramid structure. Overall, SEFormer is designed to achieve a balanced representation of local and global features, while addressing the inherent limitations of convolutional and transformer-based feature extraction.

The main contributions of this work are summarized as follows:

- We proposed SEFormer, a hybrid network that effectively integrates CNN (ResNet), SENet, and Transformer to capture both local and global features for medical image segmentation.
- We adopt a three-layer branch design, rather than a deeper four-layer structure, combined with SE-fusion and cross-attention, which reduces model parameters while maintaining high segmentation performance and computational efficiency.
- Inspired by image pyramid models, interleaved skip connections fuse multi-scale features from the convolutional and Transformer branches, enlarging the receptive field and preserving both local and global information.
- Extensive experiments on the CHASEDB dataset demonstrate that SEFormer outperforms existing methods, achieving a 3.25% improvement in segmentation accuracy.

## Related works

Medical image segmentation has witnessed significant advancements over the past decade, driven by the emergence of diverse modeling paradigms, including convolutional neural networks (CNNs), attention-based mechanisms, Transformer-based architectures, and foundation models. While each of these approaches brings unique strengths, they also present notable limitations when applied to small-scale, high-precision tasks such as retinal vessel segmentation. In the following, we provide an overview of these representative methods and highlight the key limitations that motivate the development of our proposed approach.

## CNN-based segmentation

Convolutional neural networks (CNNs) have formed the backbone of medical image segmentation due to their efficient local feature extraction and strong inductive bias for spatial patterns. Notable models include U-Net^7^, which introduced an encoder–decoder architecture with skip connections to facilitate multiscale fusion, and ResNet, which alleviated vanishing gradients through residual learning.

Variants such as SA-UNet^8^ and R2U-Net^9^ introduced attention and recurrence modules to enhance focus on lesion areas. However, CNNs inherently suffer from limited receptive fields, making them insufficient for modeling global anatomical structures. This restriction is particularly problematic in tasks requiring holistic spatial reasoning, such as blood vessel connectivity or organ boundary completion.

## Attention-enhanced CNNs

To address the locality bias of standard CNNs, several works incorporate attention mechanisms. MALUNet^10^ integrates multi-scale channel and spatial attention modules to refine feature responses, while SENet and CBAM^11^provide lightweight channel recalibration to emphasize informative filters.

While these methods enhance focus on relevant regions, they still depend on convolutional kernels for spatial encoding, which inherently restricts their capacity to capture long-range dependencies. Moreover, the manual design of attention placements can impose architectural rigidity and hinder generalization performance.

## Transformer-based methods

Transformers have emerged as powerful alternatives to CNNs, offering superior modeling of global context via self-attention. Vision Transformer (ViT) laid the groundwork, followed by Swin Transformer, which introduced shifted window attention to balance global perception and computational cost. In medical image analysis, hybrids such as TransUNet and Swin-UNet^12,13^ embed Transformer encoders within U-Net-like frameworks to enhance semantic understanding. MedT^14^addresses data scarcity by introducing gated axial-attention and a Local–Global training strategy to better preserve local details, while SegViT^15^ demonstrates that directly leveraging attention maps—rather than token features—for mask generation can yield superior segmentation performance.

Despite their strength in capturing anatomical structure, Transformer-based models face key limitations: weak local detail sensitivity, high computational demands, instability when trained on small datasets, and limited generalization or data transferability across domains. Their token-based representation often overlooks fine-grained textural cues critical in medical diagnosis. Despite their strength in capturing anatomical structure, Transformer-based models face key limitations: weak local detail sensitivity, high computational demands, instability when trained on small datasets, and limited generalization or data transferability across domains. Their token-based representation often overlooks fine-grained textural cues critical in medical diagnosis.

Recent efforts have sought to address challenges in 3D medical image analysis through architectural innovation or training strategies. UNETR^16^ adapts a pure Transformer architecture for 3D medical image segmentation, while TransBTS leverages multimodal inputs for brain tumor delineation. CoTr efficiently bridges CNN and Transformer architectures to improve segmentation performance, And beyond segmentation, TransMorph^17–19^demonstrates the versatility of Transformers in unsupervised medical image registration. To mitigate data scarcity, self-supervised pre-training of Swin Transformers has been explored in, Meanwhile, comprehensive reviews highlight the growing emphasis on multi-modality fusion and model compactness in medical vision tasks^20,21^.

## Foundation model-based approaches

With the rise of foundation models, efforts like MedSAM and H-SAM have adapted general-purpose models such as Segment Anything (SAM) for medical segmentation^22–24^. These models aim to transfer large-scale pretraining to new domains via prompt tuning or hierarchical adaptation. While effective in zero-shot or few-shot settings, such models often rely on external prompts, lack full end-to-end training, and underperform on high-resolution dense prediction tasks. Furthermore, their performance can degrade in small-scale datasets where domain-specific priors and detailed boundary localization are essential.

## Summary and motivation

In summary, existing segmentation paradigms offer partial solutions:

- CNNs provide efficient local feature encoding but fail to capture global structure;
- Transformer-based models model global context but struggle with detail preservation and computational cost;
- Foundation models generalize well but lack precision and end-to-end adaptability on task-specific data.
- Few existing methods integrate these strengths into a lightweight, effective, and data-efficient architecture suitable for medical image segmentation.

To bridge this gap, we propose SEFormer, a dual-branch architecture that combines ResNet and Swin Transformer with SENet-based channel attention. By integrating global–local information at multiple levels through feature pyramids and skip connections, SEFormer achieves accurate, efficient, and robust segmentation performance under limited data constraints.

## Methods

In this section, we will introduce the details of SEFormer method. The SEFormer method is divided into two parts: a CNN module (SE Conv) for extracting local features (which embeds a Resnet pre-trained network and SENet) and a Swin-Transformer module (SE Swin)for extracting global features (which embeds SENet as well). The image is processed through the SE Conv branch to extract local features and the SE Swin branch to extract global features. Within these branches, SENet is embedded separately. In the SE Conv branch, after embedding SENet, global features can be obtained through global average pooling (GAP) and fully connected layers. In the SE Swin branch, SENet is embedded to adaptively focus on important features by learning the weights for each channel. This helps enhance the discriminative power of local features. During the feature extraction process, we employed an image pyramid model, which processes features at three different levels to capture various receptive fields. Simultaneously, to ensure ample interaction between global and local information during the feature extraction process, we utilized the commonly used skip connection mechanism in each layer of the feature extraction process, feature information is fused and interacted to enhance overall representation.

## Overall architecture

**Fig. 1:**
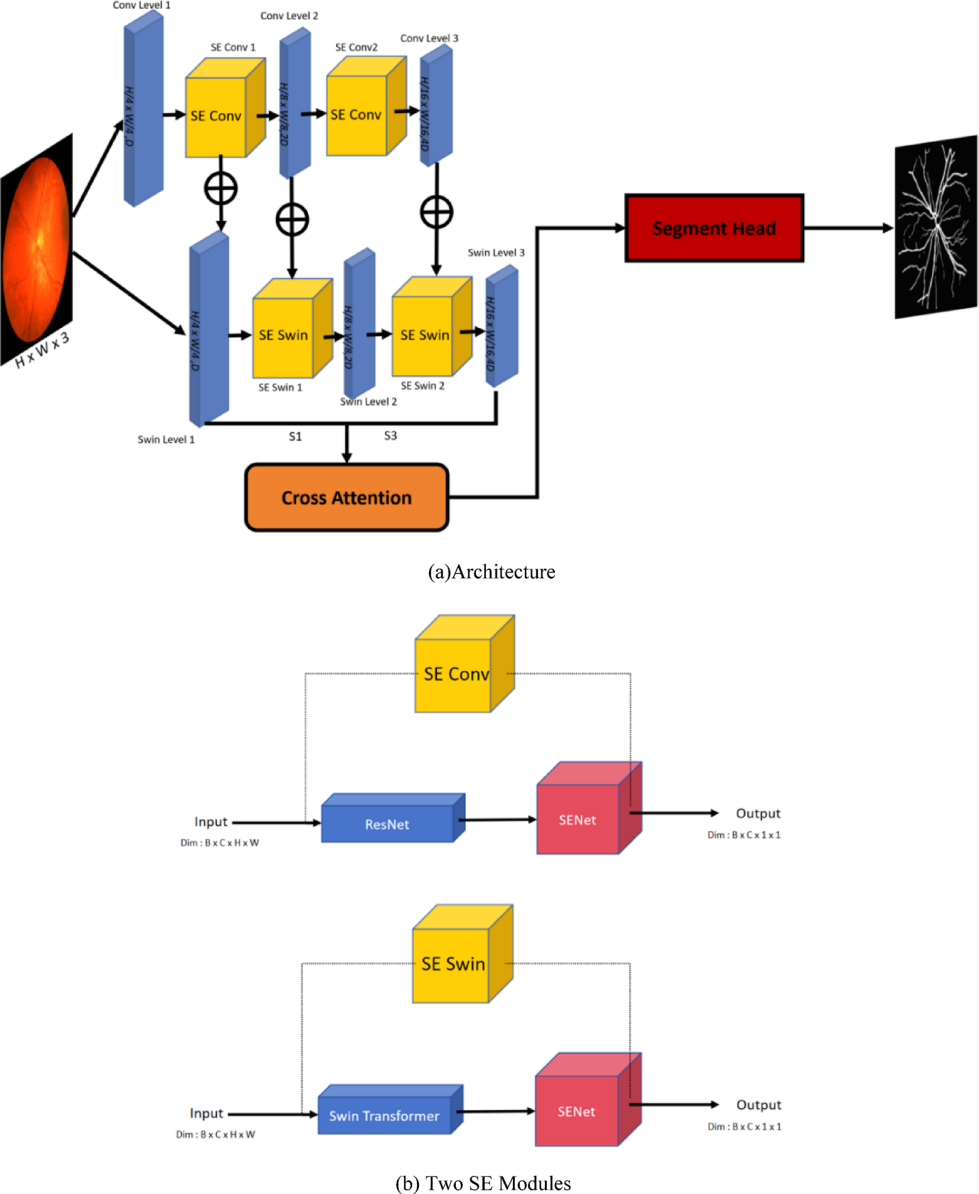
(**A**) The architecture of SEFormer method;(**B**) Two modules used to respectively enhance the extraction of local information and global information.

As shown in Fig. 1a, SEFormer comprises two major branches: SE Conv based on CNN and SE Swin based on Swin Transformer. Within SE Conv, ResNet and SENet are embedded, with ResNet primarily used for extracting local features. Simultaneously, the global pooling characteristics of SENet are utilized to gather global information, enhancing the feature extraction capability of SE Conv. Within SE Swin, Swin Transformer and SENet are integrated. Swin Transformer leverages techniques such as cross-layer connections and multi-scale feature fusion to more efficiently extract global information from the image. Subsequently, with the incorporation of SENet, a channel attention mechanism is introduced to aid in global feature modeling and enhance the network’s performance in local feature extraction. The channel attention mechanism allows the network to dynamically adjust the weights of each channel, enabling the network to focus more intently on locally significant features in different regions of the image. Based on the image pyramid model, after thoroughly extracting features as described above, in order to better preserve the rich information of the image, the features from the first convolutional level (Conv Level 1) are subjected to SE Conv for extraction, resulting in SE Conv 1. Subsequently, the result obtained by applying skip Connection to SE Conv 1 and the image data from Swin Level 1 is fed into SE Swin 1. The features extracted by SE Conv at the first level are then channel-merged to obtain Conv Level 2, which is further fused with SE Swin 1 using Skip Connection to produce Swin Level 2. Next, Swin Level 2 is passed into SE Swin 2, and the computed result is fused with Conv Level 3 using Skip Connection to yield Swin Level 3.Finally, the feature maps from Swin Level 1 and Swin Level 3 are simultaneously input into Cross Attention to better capture their inter-correlations, obtaining a more comprehensive set of image information. This approach aims to retain the original features while enhancing the information extracted after feature processing.

*2. CNN and CNNs.*

Convolutional Neural Networks (CNNs) have emerged as a powerful tool for computer vision applications.In recent years, deep learning techniques have enabled CNNs to achieve remarkable results in image recognition and image segmentation. In particular, models such as U-Net, which is designed for semantic segmentation. This kind of model exploits the advantages of stacking layers of CNNs and crafting more intricate model structures, which increase the receptive field and facilitate the extraction of richer image features. In order to solve the problem of gradient vanishing that may exist in multi-layer networks, He Kaiming and others proposed Residual Network, introducing residual mechanism. For example, ResNet introduces a residual network that alleviates the gradient-related issues of deep CNNs.

The principle of ResNet is to use skip connections to solve the problem of gradient vanishing or exploding in the training process of deep networks. Skip connections are direct connections added in the basic units of convolutional neural networks, which enable the network to learn the residual function between the input and output, rather than the mapping function directly. The residual function refers to the difference between the input and output, which is the part that needs to be learned. Skip connections can make the gradient easier to propagate in the network, thereby improving the training efficiency and performance of the network.

The U-Net network, which adopts a U-shape structure, is known for its robustness and has shown stable performance in image segmentation tasks. These models enhance the feature representation capability of deep learning models, which leads to significant improvements in the precision and efficiency of image segmentation. We present a novel model that builds on the strengths of existing models and demonstrates strong performance in the semantic segmentation of medical images. We hope that our work will inspire further research and development of models in this field.

## Swin transformer architecture

The Swin Transformer is a vision model built upon the Transformer architecture, drawing inspiration from the hierarchical design commonly employed in image processing. It has demonstrated strong performance across a wide range of visual tasks, particularly in image segmentation and object detection. Designed as a general-purpose backbone, the Swin Transformer supports various vision applications, including image classification, object detection, semantic segmentation, and video action recognition. By introducing a shifted windowing scheme and hierarchical feature representation, it effectively balances model efficiency and accuracy. Swin Transformer has achieved state-of-the-art results on numerous benchmarks, consistently outperforming traditional convolutional neural networks (CNNs) and earlier Transformer-based models.

Several key innovations contribute to the Swin Transformer’s superior performance across diverse vision tasks. It adopts a hierarchical architecture that divides an image into patches at multiple scales and applies self-attention within each layer to capture rich multi-scale feature representations. To improve efficiency, a shifted window-based self-attention mechanism is employed, which limits self-attention computation to non-overlapping local windows, significantly reducing computational and memory costs. By alternating the window partitioning across layers, the model enables cross-window information flow while preserving a global receptive field. Furthermore, an effective patch merging strategy is implemented to combine neighboring patches into larger ones, reducing the number and dimension of features, increasing the receptive field, and retaining spatial resolution.

Additionally, an effective patch merging strategy is implemented to combine neighboring patches into larger ones. This operation reduces the number and dimensionality of features, increases the receptive field, and preserves spatial resolution—further enhancing the model’s ability to process complex visual information. The architecture begins with a patch embedding module, which partitions the input image into non-overlapping patches and uses a convolutional layer to project each patch into a fixed-dimensional embedding, forming the input sequence for subsequent Transformer processing. The formula for Patch Embedding is:

1 $$X = Conv2D(I)$$

In this formula, $I$ represents the input image, and $X$ is the output feature sequence. And $Conv2D()$ is a two-dimision convolutional layer.

Patch Merging: It merges four adjacent patches into a larger patch, thereby reducing the feature map resolution, increasing the receptive field, and capturing more comprehensive image information. Specifically, Patch Merging concatenates the pixels in each patch according to their positions, and then uses a fully connected layer to map the concatenated vector to the original dimension.

2 $$Z = W_{p} X + b_{p}$$

In this context, $X$ represents the input feature sequence, $Z$ is the output feature sequence, and $W_{p}$ and $b_{p}$ are learnable parameters.

Swin Transformer Block: It consists of two sub-layers, namely Window-based Multi-head Self-Attention (W-MSA) and Multi-layer Perceptron (MLP). W-MSA divides the feature map into several non-overlapping windows, and performs self-attention within each window, thus reducing the computation cost. MLP is a feed-forward network with two fully connected layers, which enhances the feature’s non-linear expression ability.

Shifted Window Multi-head Self-Attention (SW-MSA): Similar to W-MSA, but when dividing the windows, it shifts the windows along the diagonal direction of the feature map by half of the window size, so that there are overlapping parts between adjacent windows, increasing the information interaction. SW-MSA also introduces relative position bias (Relative Position Bias), which is used to learn the relationships between different positions.

The Swin Transformer is represented by the following formula:

3 $$Y = MSA(LN(Z)) + Z$$

In this method, $Z$ represents the input feature sequence, $Y$ is the output feature sequence,$LN$ stands for Layer Normalization, and $MSA$ denotes Multi-head Self-Attention. The definitions are as follows:

4 $$MSA(Q,K,V) = Concat(H_{1} ...H_{n} ) \cdot W^{0}$$

where $Q$, $K$, and $V$ are the Query, Key, and Value matrices, $H_{i}$ is the output of the i-th head, $W^{0}$ is the linear transformation matrix for the output, Concat represents the concatenation operation. The definition of $H_{i}$ is as follows:

5 $$H_{i} = soft\max (\frac{{QW_{i}^{Q} (KW_{i}^{K} )^{T} }}{\sqrt d } + B)(VW_{i}^{V} )$$

where $W_{i}^{Q}$,$W_{i}^{K}$,$W_{i}^{V}$ are the linear transformation matrices for the i-th head, B is the relative positional bias which is denoted in Eq. 7 , and d is the dimensionality of each head.

The formula for SW-MSA is:

6 $$Y = MSA(LN(Z) + B) + Z$$

where Z represents the input feature sequence, Y is the output feature sequence, LN stands for Layer Normalization, MSA denotes Multi-head Self-Attention, and B is the relative positional bias, defined as:

7 $$B = E_{r} + E_{c}$$

where $E_{r}$ and $E_{c}$ are learnable parameters representing the relative positional biases in the row and column directions, respectively.

## SE Modules

The SE Module consists of two components: SE Conv based on CNN and SE Swin based on Swin Transformer. As shown in Fig. 1b, SE Conv incorporates ResNet to achieve enhanced feature extraction by leveraging its multi-layer convolution for richer feature representation. However, this approach still tends to overlook certain global information, especially global features within convolutional patches. To address this limitation, SE Conv embeds SENet, utilizing global average pooling. Through averaging the feature maps of each channel, the spatial dimensions of the feature maps are reduced to 1, allowing the network to better aggregate global information. Subsequently, the network enhances important features by learning channel weights obtained through this process. This mechanism contributes to improving the expressive power of the network, enabling it to better adapt to the feature distribution of input data. Consequently, it results in enhanced performance in tasks such as image segmentation.SE Module uses the dual-branch design to fully extract the global and local information in the image, and better achieve the image segmentation task.

### SE Conv module

The SE Conv module employed a pre-trained ResNet model for feature extraction is utilized, though alternative backbone networks can also be integrated for this purpose. ResNet adopts Residual Blocks, which effectively mitigate the vanishing gradient problem encountered when training very deep networks. As shown in Fig. 1b, ResNet is cascaded with SENet. The input has dimensions of B × C × H × W, and the final output is compressed to B × C × 1 × 1, facilitating feature fusion in subsequent stages and serving as input to the Cross Attention module. Meanwhile, the features extracted by SE Conv are fused with outputs from SE Swin and Swin Level via skip connections. This strategy effectively preserves both global and local information throughout the feature extraction process.

### SE Swin module

**Fig. 2:**
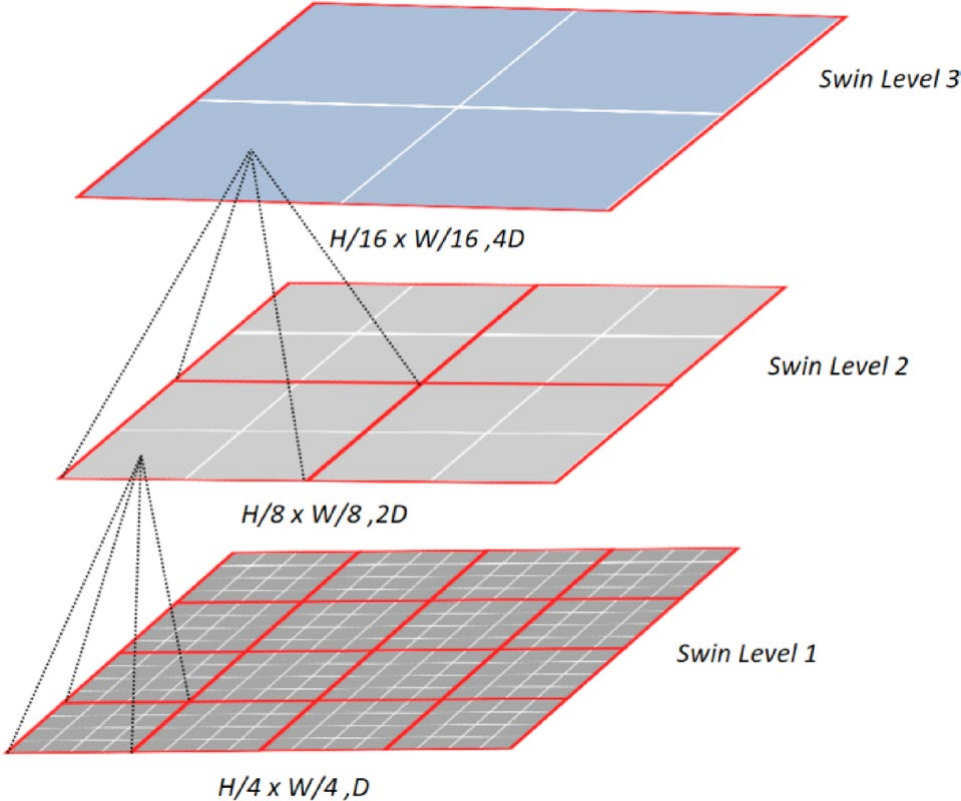
Swin transformer model.

As shown in Fig. 2, Swin Transformer adopts a hierarchical structure by decomposing the input image into a series of non-overlapping image patches, with each patch treated as a “token”. This hierarchical structure helps enhance the model’s ability to process large-scale images. Swin Transformer can reduce the computational cost of attention. It introduces a novel attention mechanism that divides the attention window into multiple sub-windows, allowing for displacement operations between the windows. This design aims to decrease computational complexity while maintaining a larger receptive field for capturing long-range dependencies. However, relying solely on this approach compromises the extraction of local features. To address this limitation, the SE Swin module is introduced in this study.

The introduction of the Squeeze-and-Excitation mechanism facilitates global information modeling while simultaneously enhancing the network’s ability to extract local features from images. The role of Excitation step is to adaptively learn weights for each channel, enabling the network to focus more selectively on crucial features. This is particularly beneficial for extracting local information in images. Here, we cascade Swin Transformer with SENet. The input image is adjusted to the same size as the SE Conv, undergoing similar adjustments in terms of size and channels. In Swin Level 1, we process an image with dimensions D and size H/4 × W/4. After feature extraction with SE Swin, the dimensions become 2D, and the size becomes H/8 × W/8, resulting in Swin Level 2. Subsequently, we continue adjusting the image size and dimensions, obtaining H/16 × W/16, with dimensions becoming 4D, ultimately achieving Swin Level 3.

## Cross attention

Cross-attention is an attention mechanism commonly employed to handle tasks involving two distinct sequences, where elements of one sequence need to focus on relevant information from another sequence. It is widely utilized in the fields of natural language processing and computer vision^25^.

**Fig. 3:**
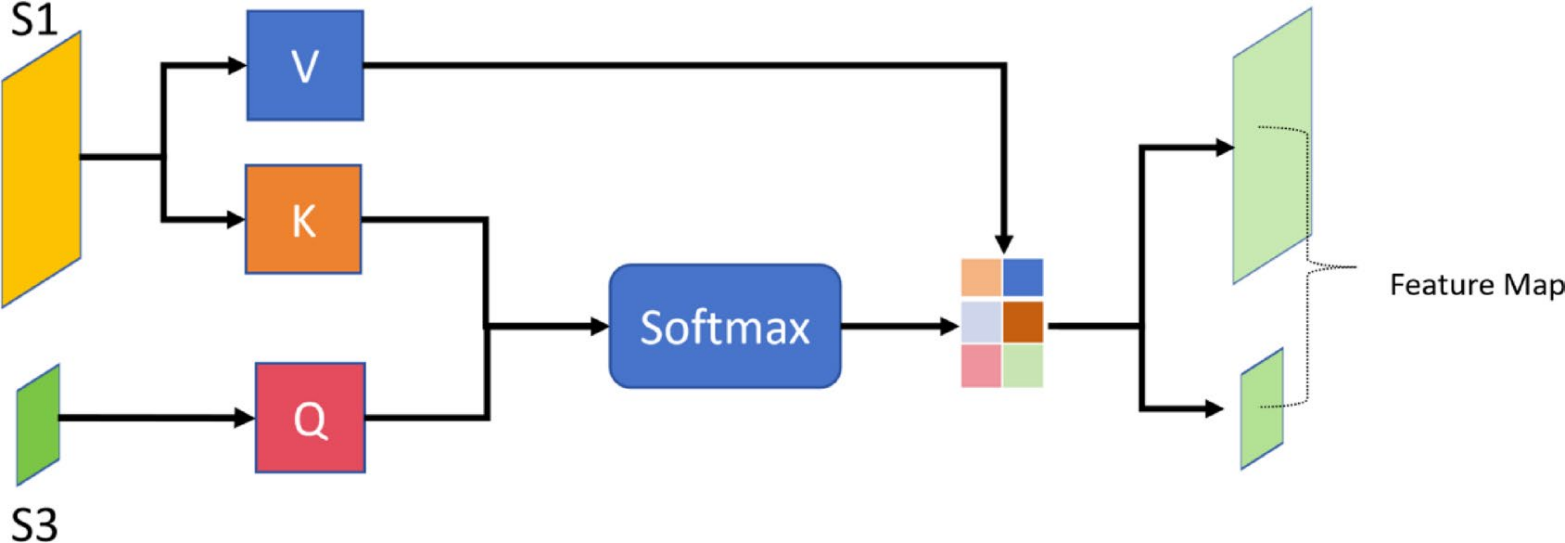
Cross attention.

As shown in Fig. 3, the working principle of cross-attention involves defining Query, Key, and Value, calculating weights, and obtaining the final output through weighted summation. Its main purpose is to enable the model to better focus on information from another sequence that is relevant to the current position when processing one sequence, thereby enhancing the model’s performance in related tasks.

Based on the feature extraction results using the SE Module in the image pyramid model, the top-level representation $S_{3}$ and the bottom-level representation $S_{1}$ are obtained. These two representations serve as the input for Cross Attention, allowing for a more comprehensive fusion of feature information. Specifically,$S_{3}$ is used to generate the Query ($Q$), while $S_{1}$ is used to generate the Key ($K$) and Value ($V$).In the end, we obtained two feature maps of the same size as $S_{1}$ and $S_{3}$.

When using two different hierarchical feature maps,$S_{1}$ and $S_{3}$, for cross-attention, where $S_{3}$ generates Query $Q$ and $S_{1}$ generates Key $K$ and Value $V$, the formulas can be expressed as:

8 $$Cross\_Attention\_Output(q_{i} ) = \sum\limits_{j = 1}^{m} {Attention\_Weights(q_{i} ,k_{j} ) \cdot v_{j} }$$

where, Formula 8:

9 $$Attention\_Weights(q_{i} ,k_{j} ) = \frac{{\exp (Attention(q_{i} ,k_{j} ))}}{{\sum {_{j = 1}^{m} \exp (Attention(q_{i} ,k_{j} ))} }}$$

and the attention mechanism $Attention(Q_{i} ,K_{j} )$ can be formulated using a scaled dot-product attention, for example:

10 $$Attention(Q_{i} ,K_{j} ) = \frac{{Q_{i} \cdot K_{j} }}{{\sqrt {d_{k} } }}$$

Here,$d_{k}$ is the dimensionality of Query($q_{i}$) and Key($k_{j}$).

## Results

### Dataset

We evaluated the proposed SEFormer model on three widely-used retinal vessel segmentation datasets: CHASE_DB1, DRIVE, and STARE. These datasets cover diverse patient demographics, imaging conditions, and vessel characteristics, enabling a comprehensive assessment of model performance and generalization.

CHASE_DB1 is a high-resolution dataset comprising 28 RGB fundus images, each with a resolution of 999 × 960 pixels. The images were acquired from the left and right eyes of 14 school-aged children using a Nidek NM-200-D fundus camera. Each image is annotated by two expert graders. The first annotation is typically used as the ground truth in segmentation tasks. This dataset presents challenges such as variable illumination, fine vessel structures, and low contrast.

DRIVE consists of 40 color retinal images with a resolution of 768 × 584 pixels. The images were obtained from a diabetic retinopathy screening program in the Netherlands. It is divided into a standard training set (20 images) and testing set (20 images), each with ground truth annotations provided by two independent observers. The dataset includes both normal and pathological cases, and is widely used as a benchmark for vessel segmentation performance and cross-domain generalization.

STARE (Structured Analysis of the Retina) contains 20 fundus images with a resolution of 700 × 605 pixels. These images were collected from both healthy and diseased subjects, and manually annotated by two experts with different grading criteria. In our experiments, we used the first observer’s annotation as the reference. STARE poses unique challenges due to the presence of abnormal vessel structures and varied imaging conditions.

In our setup, CHASE_DB1 was used as the primary dataset for training and evaluating segmentation performance, while DRIVE and STARE were employed to assess the cross-dataset generalization capability of SEFormer. All datasets were preprocessed and augmented using a unified pipeline, as detailed in Section "Training details". The CHASE_DB1 dataset, which contains 28 fundus images, was randomly split into 60% (17 images) for training, 20% (6 images) for validation, and 20% (5 images) for testing.

The same preprocessing and partitioning strategy was consistently applied across all models. For the DRIVE dataset, we adopted the official split, with 20 images for training and 20 for testing. For the STARE dataset, 80% of the images (16) were randomly selected for training and the remaining 20% (4) for testing during the cross-dataset generalization evaluation.

## Training details

All experiments were implemented in PyTorch 1.13 and conducted on a workstation equipped with an NVIDIA RTX 3090 GPU (24 GB VRAM), 256 GB RAM, and an Intel Xeon 6226R CPU. The Python environment was managed with conda, and key dependencies included Albumentations for data augmentation, NumPy for data handling, and Matplotlib for visualization.

## Model initialization

All models, including SEFormer and baselines (U-Net, SA-UNet, MALUNet, MedSAM, and H-SAM), were trained from scratch using random weight initialization (He normal). No pre-trained weights or transfer learning were used to ensure a fair comparison. All models used the same backbone depth and comparable number of parameters wherever applicable.

## Optimization

We used the Adam optimizer with default β1 = 0.9 and β2 = 0.999, a weight decay of 1e-5, and an initial learning rate of 1e-4. The batch size was set to 4, and the number of training epochs was fixed at 200. A StepLR learning rate scheduler was used to reduce the learning rate by a factor of 0.1 if the validation loss did not improve for 10 consecutive epochs. All models were trained using mixed-precision (fp16) mode via PyTorch AMP to accelerate training and reduce memory usage.

## Loss function

The loss function was defined as a combination of Binary Cross-Entropy (BCE) and Dice Loss to jointly optimize pixel-level accuracy and region-level overlap. The composite loss is given by:

11 $$L_{total} = \alpha \cdot L_{BCE} + (1 - \alpha ) \cdot L_{Dice} \begin{array}{*{20}c} {} & {} \\ \end{array} ,\alpha = 0.5$$

Dice Loss is particularly effective in handling class imbalance, which is common in medical segmentation datasets such as CHASE_DB1 ,DRIVE and STARE.

## Data loading and augmentation

All input images were resized to 512 × 512 and normalized to the [0, 1] range. To enhance model generalization, on-the-fly data augmentation was applied during training using the Albumentations library. The augmentation pipeline included random horizontal and vertical flips, rotations within ± 10°, brightness and contrast adjustments, as well as elastic deformation and Gaussian noise (applied with a probability of 0.3). No augmentations were applied during the validation and testing phases.

## Training and evaluation protocol

Each epoch consisted of a complete pass through the training dataset, followed by validation on a fixed hold-out set. Model checkpoints were saved based on the highest Dice coefficient on the validation set. To prevent overfitting, early stopping was triggered if the validation Dice score did not improve over 20 epochs.

At the conclusion of training, the model checkpoint yielding the highest Dice score on the validation set was selected for final evaluation. All reported results (Accuracy, mIoU, DSC) were computed on the test set using a consistent evaluation metrics across all models.

## Segmentation results

### Segmentation on CHASE_DB1 dataset

**Fig. 4:**
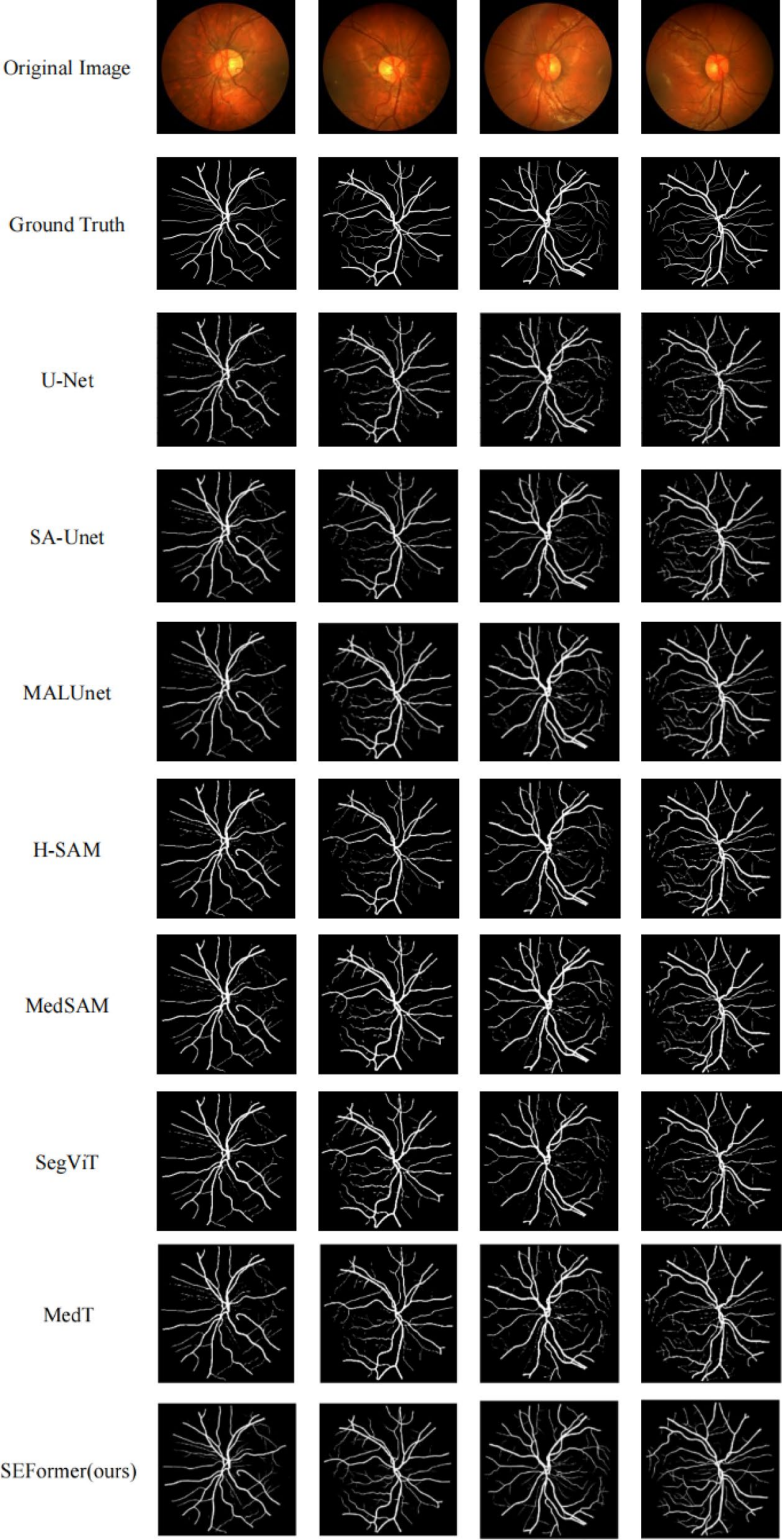
Different method for chase_DB1 dataset segmentation.

In our rigorous exploration of the CHASE_DB1 dataset, we conducted a comprehensive comparison of segmentation methods, focusing on SEFormer, U-Net, SA-UNet, and MALUNet. To facilitate visual assessment, we present representative results comprising the original image, ground truth, and the segmentation outputs of each model, with particular emphasis on the superior performance of SEFormer. These results are illustrated in Fig. 4. The first column shows the original images, the second column shows the ground truth, and the following columns show the segmentation results of U-Net, SA-UNet, MALUNet,H-SAM,MedSAM,SegVit,MedT and SEFormer (ours), respectively.

A discerning scrutiny of the visual representations reveals a clear hierarchy in segmentation prowess, with SEFormer emerging as the unequivocal frontrunner. SEFormer’ s segmentation outcomes outshine those of U-Net, SA-UNet, MALUNet, H-SAM, MedSAM,SegVit,MedT particularly evident in the intricate delineation of capillary structures. This nuanced success is indicative of SEFormer’ s exceptional ability to capture and reproduce fine details and intricacies, a crucial aspect often challenging for other methodologies.

Among the methodologies scrutinized, U-Net exhibits the least favorable segmentation results, displaying a discernible discrepancy when compared to the Ground Truth. In contrast, both SA-UNet and MALUNet perform similarity at an intermediate level, yet their segmentation precision lags behind that of SEFormer. The visual results highlights SEFormer’ s high fidelity in replicating the groundtruth for capillary segmentation, demonstrating its robustness in capturing intricate features that are frequently missed or distorted by alternative methodologies.

**Fig. 5:**
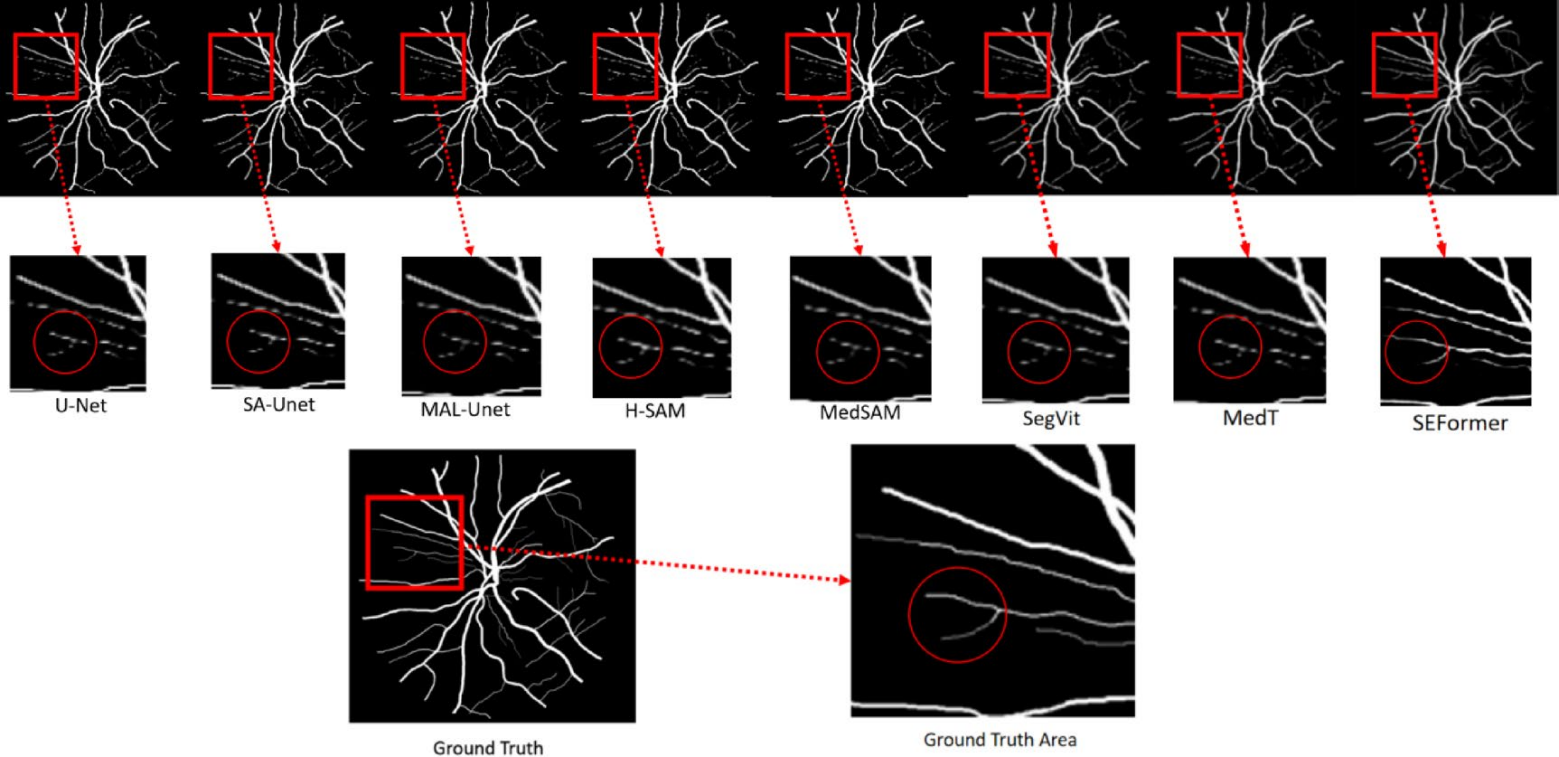
Details of different partitioning methods comparison.

To provide a comprehensive and detailed analysis of the segmentation results, a thorough horizontal comparison was performed on the same image, as shown in Fig. 5. The first row represents the segmentation results of different methods on the same image. The second row illustrates the comparison of local details, while the third row shows the Ground Truth labels in the corresponding regions. Focus was placed on a localized region within the red box, where careful examination yielded valuable insights, especially regarding vascular segmentation performance.

In the domain of vascular segmentation, SEFormer demonstrated superior performance, exhibiting exceptional capability in capturing fine-grained details—such as subtle branches—within the complex vascular structures of the eyeball. This observation underscores the method’s remarkable ability to discern and delineate the fine anatomical structures that are crucial for an accurate representation of vascular patterns.

Furthermore, an essential characteristic of the SEFormer approach lies in its concerted effort to preserve image features to the utmost extent. This commitment to feature preservation is clearly reflected in its intricate and comprehensive segmentation of blood vessels, emphasizing the method’s dedication to capturing even the most delicate aspects of the underlying anatomical structures.

In stark contrast, while methodologies such as U-Net, SA-U-Net, SegViT, MedT,H-SAM, MedSAM, ,SegVit,MedT and MALUNet demonstrate commendable accuracy in extracting the primary vascular skeleton during segmentation tasks, they exhibit noticeable shortcomings. These limitations often manifest as interruptions in the continuity of vascular branches and terminals, highlighting a reduced capability in preserving finer structural details. Such deficiencies become particularly significant in applications where high segmentation fidelity is imperative, especially in medical imaging scenarios that support clinical diagnosis.

Fundamentally, the horizontal comparison of segmentation outcomes not only underscores the superior performance of SEFormer in discerning intricate vascular details but also underscores the method’s steadfast dedication to preserving essential image features. This nuanced analysis enriches our comprehension of the distinctive strengths and limitations inherent in each methodology, thereby fostering a more informed decision-making process when choosing an appropriate approach for specific image segmentation tasks.

**Fig. 6:**
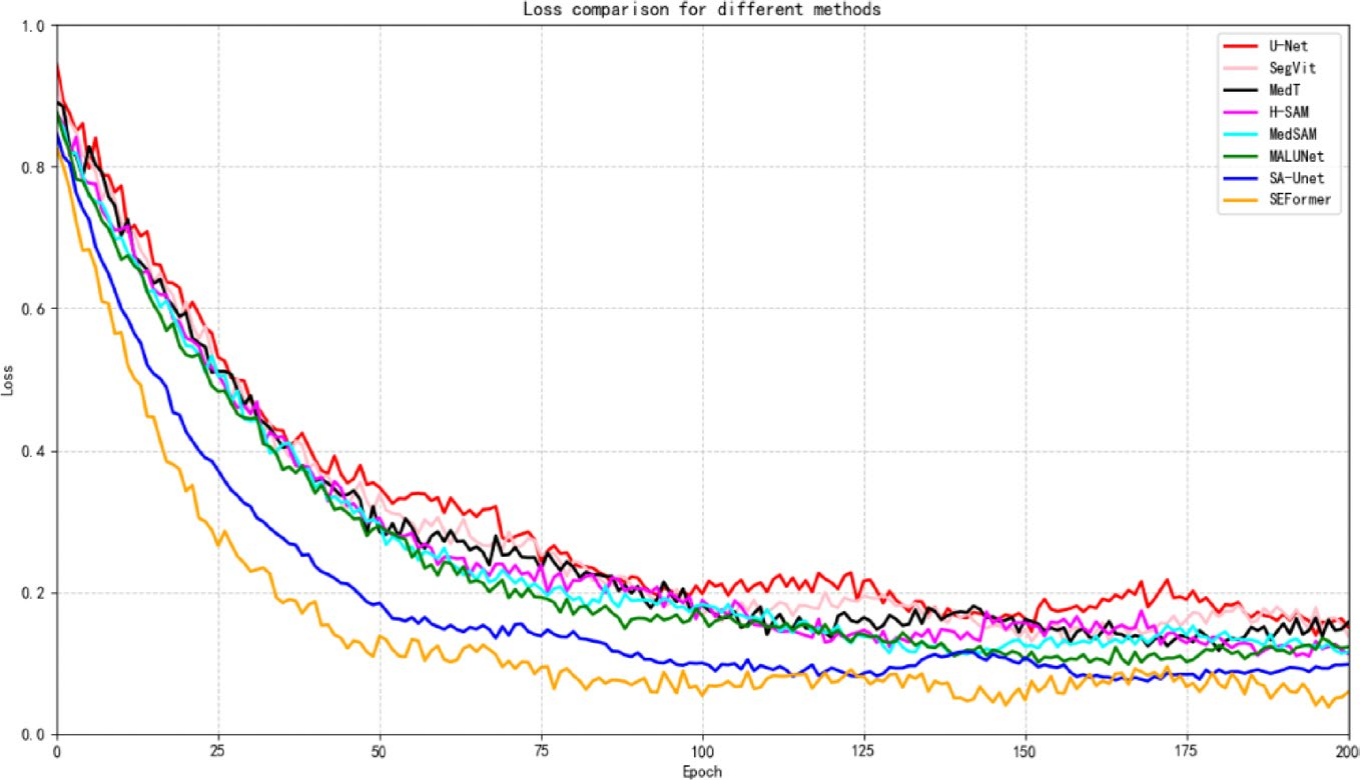
Loss comparison for different methods.

As shown in Table 1, our method demonstrates outstanding performance across key segmentation metrics, achieving a mean Intersection over Union (mIoU) of 90.76, Accuracy of 97.71, and Dice Similarity Coefficient (DSC) of 94.19. These results reflect the model’s high precision in delineating object boundaries, accurate pixel-wise classification, and strong spatial agreement with the ground truth. The high mIoU score indicates substantial overlap between predicted masks and annotations, while the Accuracy and DSC scores highlight the method’s ability to maximize true positives and minimize both false positives and false negatives. Collectively, these metrics provide a comprehensive evaluation of segmentation performance.In addition to quantitative superiority, our method exhibits faster convergence and lower training loss, as illustrated in Fig. 6, underscoring its efficiency in both learning and computation—an advantage for time-sensitive applications^14,15^.

**Table 1:** The result on the chase_DB1 dataset.

Methods	Accuracy↑	Precision↑	mIOU↑	DSC↑
U-Net	92.88	89.23	81.12	88.11
SA-UNet	93.96	**96.92**	85.33	89.31
MALUNet	91.62	91.11	87.11	83.87
H-SAM	90.22	90.03	83.26	82.09
MedSAM	91.29	87.07	81.01	80.85
SegVit	90.08	88.17	80.05	79.63
MedT	89.77	86.35	81.09	80.13
SEFormer(ours)	**97.71**	96.18	**90.76**	**94.19**

Overall, the results in Table 1 validate the robustness and effectiveness of the proposed approach, establishing it as a competitive solution for high-precision image segmentation tasks.

In Fig. 6, the distinctive loss profiles of various methodologies are presented, with U-Net depicted in red, MaLUNet in green, SA-UNet in blue, H-SAM in purple, MedSAM in cyan,SegVit in pink,MedT in black, and SEFormer in brown. The x-axis shows the number of epochs, and the y-axis shows the loss value. The curves represent the loss of U-Net, SA-UNet, MALUNet,H-SAM,MedSAM,SegViT, MedT and SEFormer (ours), respectively. Strikingly, the U-Net curve occupies the highest trajectory, succeeded by H-SAM,MedSAM, MaLUNet ,MedT,SegVit and SA-UNet, while SEFormer consistently maintains the most favorable loss trajectory. Notably, SEFormer initiates training with a remarkably low loss, attesting to its exceptional efficacy right from the initial epochs, and further establishes itself as the fastest converging method.

A detailed examination of the training curves reveals that U-Net exhibits a slow and gradual convergence, indicating limited training efficiency. In contrast, MALUNet converges more quickly than U-Net but remains slower than both SA-UNet and SEFormer. SA-UNet demonstrates a moderate convergence speed, while SEFormer stands out for its rapid convergence from the early stages of training.

Notably, SEFormer not only achieves the lowest training loss but also reaches this optimal state significantly earlier than all other methods—converging effectively by the 180th epoch. This reflects SEFormer’s efficiency in capturing complex patterns and features within the dataset.

As illustrated in Fig. 6, these trends provide meaningful insight into the convergence behavior of different models. SEFormer clearly leads with both faster convergence and minimal loss, reinforcing its superiority in efficient and accurate image analysis.

### Generalization on external datasets

To evaluate the generalizability of the proposed SEFormer model, we conducted additional experiments on two external datasets: DRIVE and STARE. Both datasets differ from CHASE_DB1 in terms of acquisition settings, image resolution, and population characteristics, making them suitable for cross-dataset validation. Importantly, the SEFormer model was trained exclusively on CHASE_DB1 and directly applied to DRIVE and STARE without any fine-tuning or domain adaptation.

The quantitative results, summarized in Table 2, demonstrate that SEFormer consistently achieves high Dice coefficients and mIoU scores on both datasets, confirming its robustness under distribution shift.

**Table 2:** The result on the DRIVE and STARE dataset.

Dataset	Accuracy (%)	Dice (%)	mIoU (%)
DRIVE	96.84	89.62	84.77
STARE	96.12	87.45	82.31

**Fig. 7:**
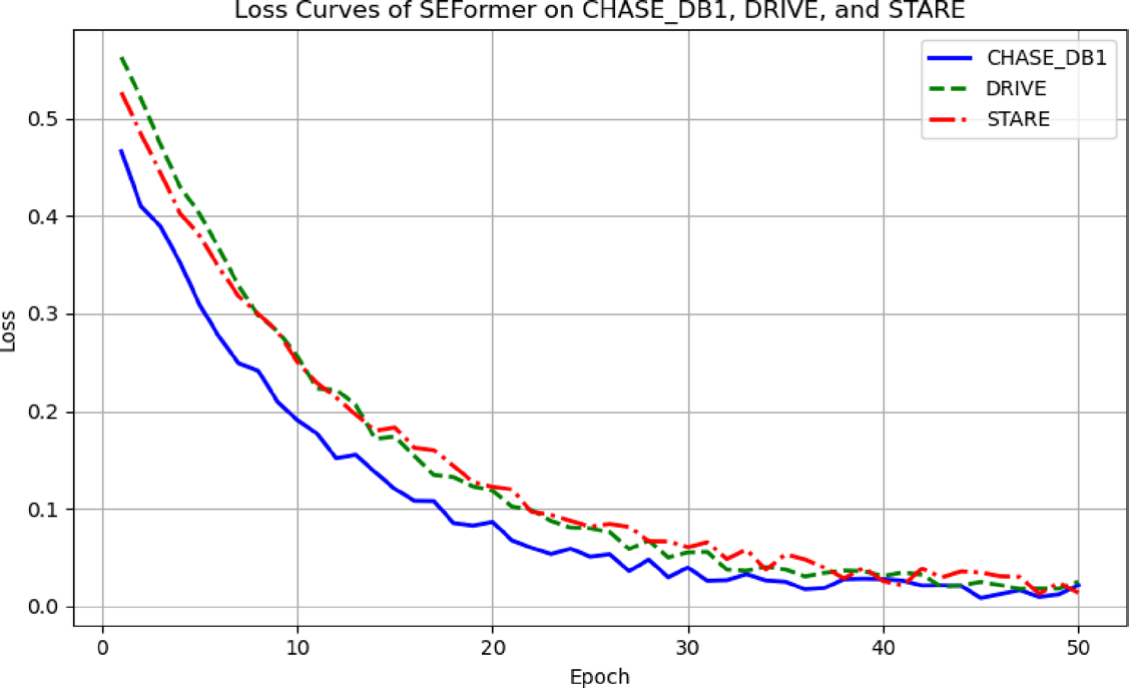
Loss curves of SEFormer evaluated on Chase_DB1, drive and stare datasets.

In addition to the quantitative metrics, the loss curves on the DRIVE and STARE datasets are presented in Fig. 7. The results demonstrate that SEFormer converges stably on both datasets, despite the absence of any retraining or domain-specific adaptation.

As illustrated in Fig. 7, the SEFormer model demonstrates a smooth and rapid convergence on all three datasets. The loss decreases steadily within the first 30 epochs and plateaus without fluctuation, indicating effective learning and strong training stability. Notably, the convergence patterns on DRIVE and STARE, which were not used during training, further confirm SEFormer’s robustness and generalization capability under cross-domain scenarios.

**Fig. 8:**
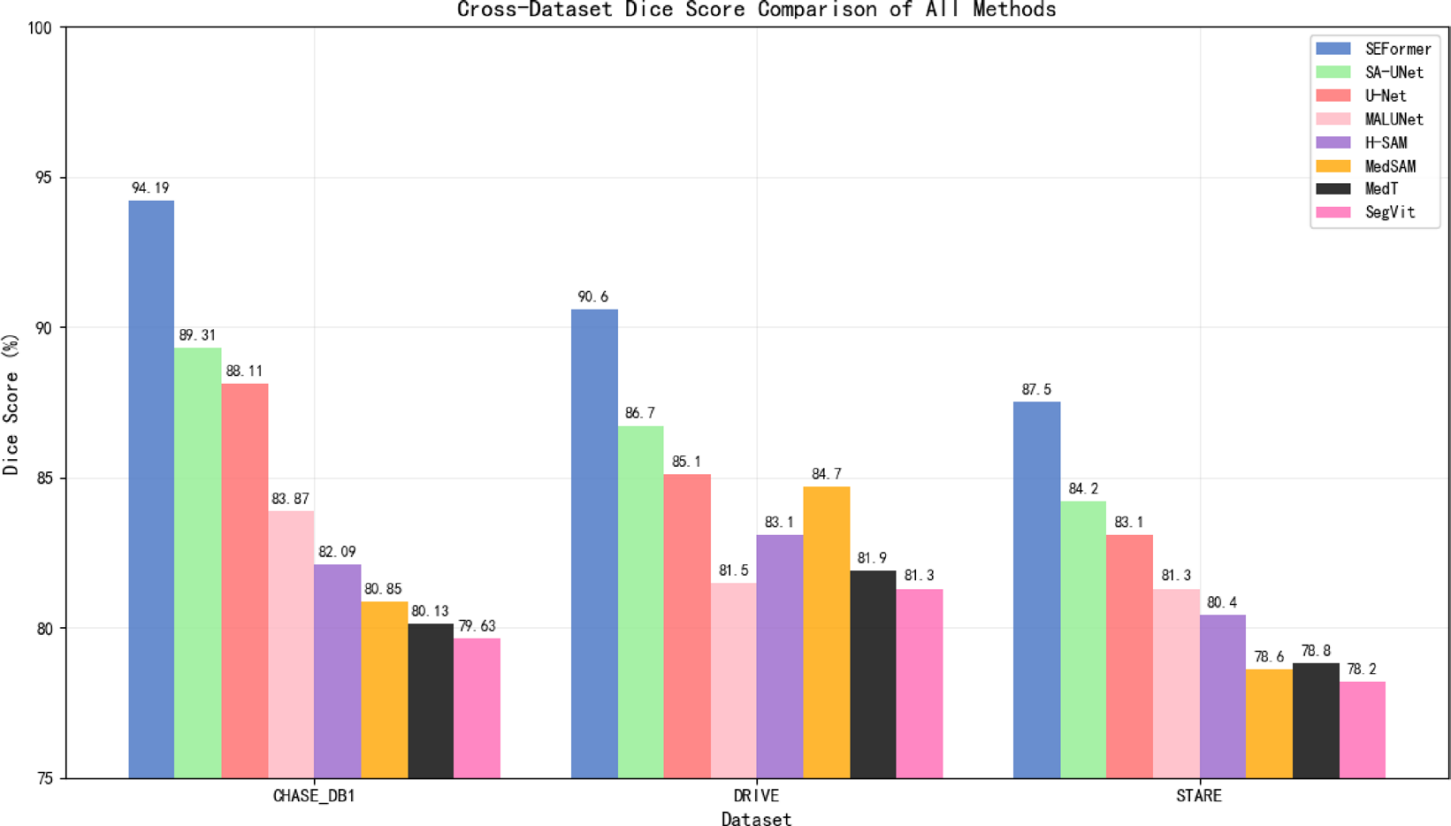
Cross-dataset dice score comparison of all methods.

As illustrated in Fig. 8, SEFormer achieves the highest Dice score on all three datasets, demonstrating superior region-wise segmentation accuracy. Notably, the performance gap is more pronounced on external datasets (DRIVE and STARE), confirming SEFormer’s strong generalization ability. SA-UNet also shows competitive results, but lags behind SEFormer, particularly on unseen domains.

**Fig. 9:**
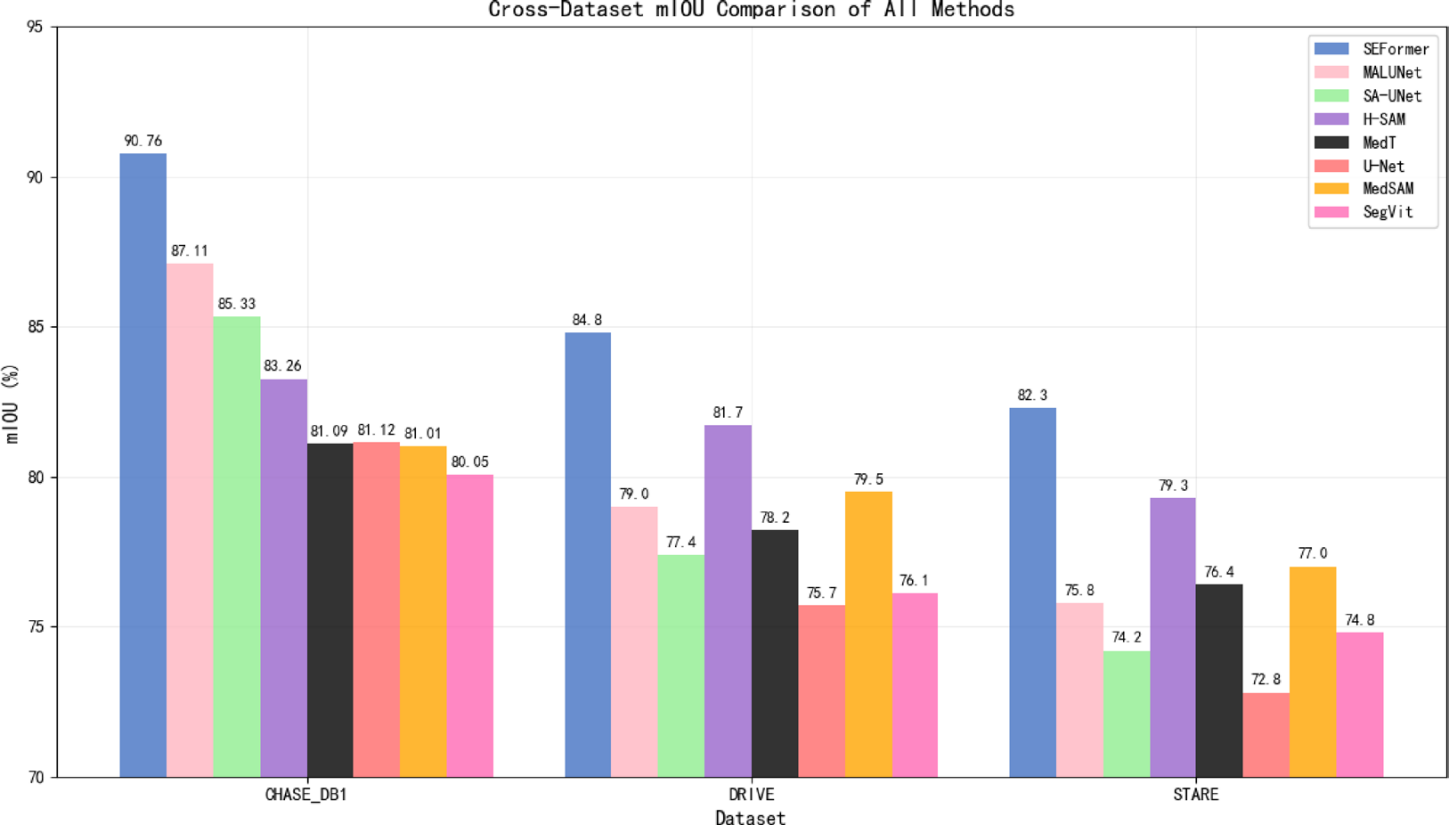
Cross-dataset mIoU comparison of all methods.

In terms of mIoU, as depicted in Fig. 9, SEFormer maintains a significant lead over all baseline methods, particularly on the CHASE_DB1 dataset (90.76%) and generalization datasets (84.8% on DRIVE and 82.3% on STARE). These results indicate the model’s robustness in capturing complete structures with minimal false positives or fragmentation. Compared to MedSAM and H-SAM, SEFormer shows a clear advantage in both precision and generalization.

### Complexity and efficiency analysis

To further address the concern regarding model complexity, we report the number of parameters, FLOPs, model size, and inference time of SEFormer and representative segmentation models in Table 3.

**Table 3:** Performance and efficiency comparison of medical image segmentation models.

Model	Params (M)	FLOPs (G)	Size (MB)	Time (ms)
U-Net	30	65	40	12
SA-UNet	35	70	57	18
MALUNet	32	75	55	16
H-SAM	91	80	140	33
MedSAM	95	90	132	28
SegViT	80	95	110	30
MedT	86	100	108	23
SEFormer	81	85	99	20

Table 3 compares the complexity and efficiency of representative segmentation models. SEFormer contains 81 M parameters and 85G FLOPs, with a model size of 99 MB and an inference time of 20 ms. Although it is not the smallest in parameters (e.g., U-Net, 30 M) or the fastest in inference (e.g., U-Net, 12 ms), SEFormer is more efficient than recent Transformer-based models such as SegViT (80 M, 95G, 110 MB, 30 ms) and MedT (86 M, 100G, 108 MB, 23 ms)^14,15^. To further control complexity, SEFormer adopts a three-layer design in each branch, rather than deeper stacks, which effectively reduces the parameter scale while preserving representational capacity. These results indicate that the integration of SENet and cross-attention does not incur excessive computational burden, and that SEFormer achieves a favorable balance between model complexity and inference efficiency while maintaining competitive segmentation performance.

### Ablation study

We conducted ablation experiments to evaluate the contribution of each component in SEFormer (Table 4).

**Table 4:** SEFormer ablation experiments.

Experiment	ResNet branch	Swin branch	Fusion	Cross attention	Params (M)	FLOPs (G)	Size (MB)	Dice (%)	Inference time (ms)
Full SEFormer	✔	✔	SE-fusion	✔	81	85	99	91.2	20
SE Swin	✖	✔	SE-fusion	✔	54	60	68	88.5	16
SE Conv	✔	✖	SE-fusion	✔	58	62	70	87.3	15
Skip-Connection	✔	✔	Add/Concat	✔	69	83	82	89.0	19
W/O Cross-Attention	✔	✔	SE-fusion	✖	65	72	84	76.0	19

As shown in Table 4 ,removing either the SE Conv branch or the SE Swin branch reduces parameters (81 M → 54–58 M) and FLOPs (85G → 60–62G), while decreasing Dice from 91.2% to 87.3–88.5%, indicating that the two branches provide complementary representations. Replacing SE-fusion with a simple skip-connection lowers complexity (69 M, 83G) but also slightly reduces Dice to 89.0%, confirming the effectiveness of the proposed fusion strategy.

Eliminating the cross-attention mechanism reduces parameters (81 M → 65 M), FLOPs (85G → 72G), and inference time (20 ms → 19 ms), yet results in a substantial drop in Dice to 76.0%. This demonstrates that cross-attention is crucial for integrating local and global features. Overall, the full SEFormer achieves the best balance between segmentation accuracy and computational efficiency, with each component making a meaningful contribution to performance.

## Conclusion

We propose SEFormer, a hybrid neural architecture that effectively combines convolutional operations and attention mechanisms for medical image segmentation. By integrating SENet for channel calibration, residual convolutions for capturing local patterns, and Swin Transformer blocks for modeling global dependencies, SEFormer achieves a compact yet expressive representation of anatomical structures. Evaluated on three benchmark datasets—CHASE_DB1, DRIVE, and STARE. SEFormer consistently outperforms CNN-based baselines as well as recent foundation models such as H-SAM ,SegVit,MedT and MedSAM in terms of Dice score and mIoU. The model demonstrates faster convergence and superior generalization without the need for large-scale pretraining or prompt tuning, underscoring its potential for practical clinical deployment.

Although SEFormer achieves competitive performance and efficiency, several limitations remain. The reliance on Transformer blocks still imposes non-negligible computational overhead. The sensitivity to small datasets may affect model stability and generalization in real-world clinical scenarios. Furthermore, the current design focuses on 2D segmentation; its extension to 3D volumetric data and multi-modal inputs requires further exploration.

## Data Availability

The datasets used in this study are all publicly available and widely adopted in the retinal vessel segmentation community. **CHASE_DB1:** The CHASE_DB1 dataset was collected as part of the Child Heart and Health Study in England (CHASE), a multi-ethnic population-based cohort of school-aged children. It contains high-resolution retinal fundus images annotated by two medical experts. The dataset is publicly available via Kingston University’s research data repository: https://researchdata.kingston.ac.uk/96/. **DRIVE:** The Digital Retinal Images for Vessel Extraction (DRIVE) dataset consists of retinal images obtained from a diabetic retinopathy screening program in the Netherlands. It includes 40 color fundus images with expert annotations and is publicly accessible at: https://drive.grand-challenge.org/. **STARE:** The Structured Analysis of the Retina (STARE) dataset contains 20 retinal images captured from both healthy and diseased subjects. Each image is labeled by two ophthalmologists. It is publicly available at: https://cecas.clemson.edu/ ~ ahoover/stare/. No proprietary or non-public datasets were used in this study. All experiments were conducted on openly accessible resources to ensure reproducibility.
